# Supplementary material for: A brain-to-small intestine circuit mediates morphine-induced constipation in male mice
Source: Nat Commun. 2025 Dec 23;17:1023. doi: 10.1038/s41467-025-67765-7 (PMC12847740; doi:10.1038/s41467-025-67765-7)
Supplement: Supplementary file 1 — Supplementary Information [file 41467_2025_67765_MOESM1_ESM.pdf]

Supplemental figures and tables

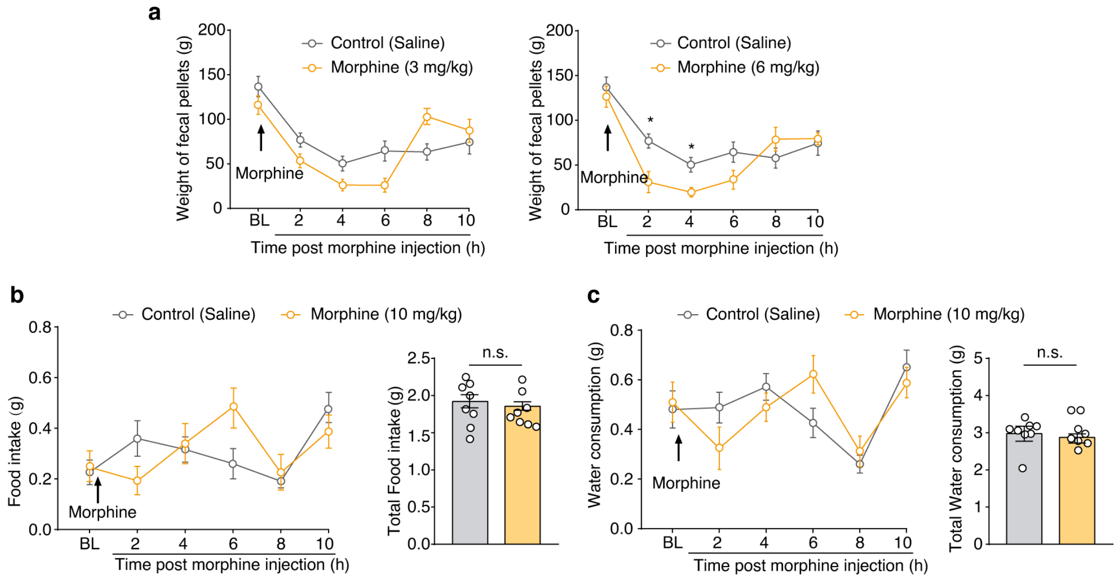

Supplementary Fig. 1 | Food intake and water consumption in mice after morphine injection. a, Weight of feces in mice following morphine injection at doses of 3 mg/kg (left) and 6 mg/kg (right) (3 mg/kg: n = 10, $F_{1,18}$ = 3.867, P = 0.0649; 6 mg/kg: n = 10, $F_{1,18}$ = 4.554, P = 0.0260 for 2 h, P = 0.0409 for 4 h). **b**, Food intake at various time points (left) and total food intake (right) following morphine injection (n = 8, $F_{1,14}$ = 0.0093, P = 0.9245 for food intake; P = 0.9591 for total food intake). **c**, As indicated in panel **b**, but for water consumption after morphine injection (n = 8, $F_{1,14}$ = 0.0419, P = 0.8408 for food intake; P = 0.2786 for total food intake). Significance was assessed by two-way repeated-measures ANOVA with post hoc comparison between groups (**a-c**) and Mann-Whitney U test (**b** and **c**). The data are presented as the mean ± SEMs. *P < 0.05; n.s., not significant. See also Supplementary Table S1. Source data are provided as a Source Data file.

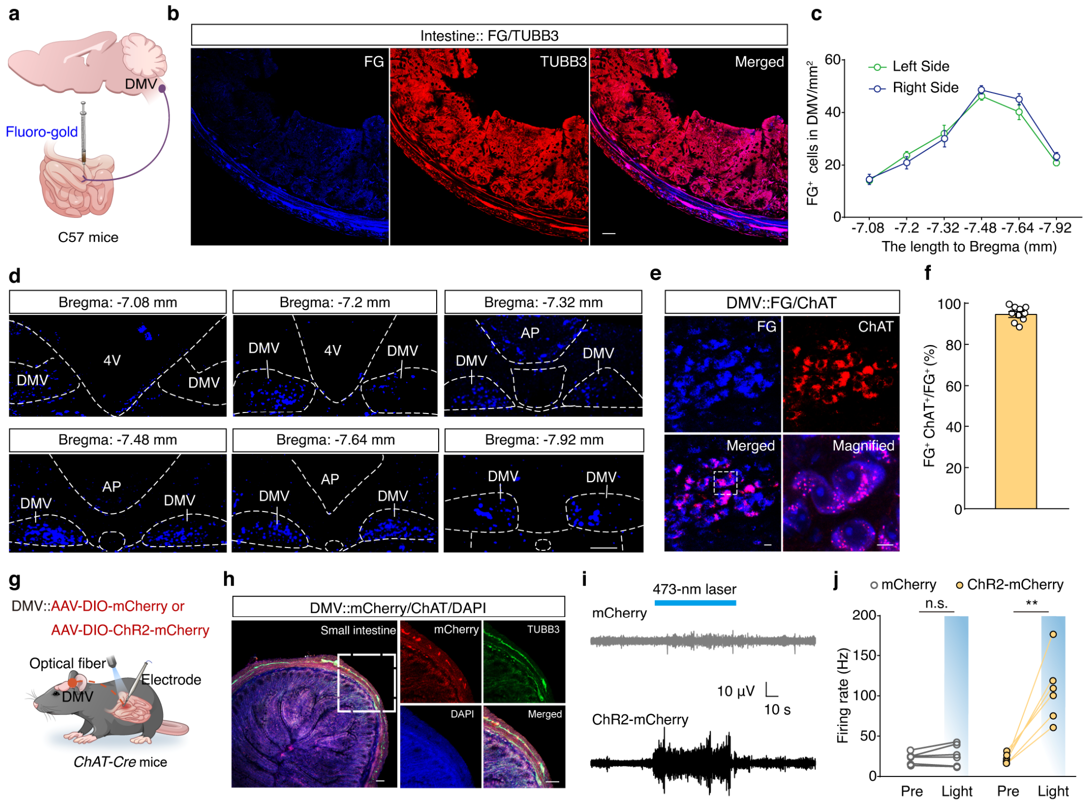

Supplementary Fig. 2 | DMV^{Ach} neurons directly project to the small intestine. a, Schematic diagram for Fluoro-Gold (FG) injection into the small intestinal wall. **b**, Representative images showing co-localization of the FG$^+$ and TUBB3 antibody signals in the small intestinal wall. Scale bar, 50 μm. **c**,**d**, Representative images (**d**)and summary data (**c**) showing FG expression in the dorsal motor nucleus of the vagus (DMV) at 7 days after FG injection. Scale bar, 100 μm (n = 5, $F_{1,8}$ = 0.5010, P = 0.4991). **e**,**f**, Representative images (**e**) and summary data (**f**) for FG$^+$ neurons colocalized with a choline acetyltransferase (ChAT, a marker for cholinergic neurons)-specific antibody in the DMV. The magnified image in the lower right depicts the area shown in the white box of the DMV. Scale bars, 10 μm. **g**, Schematic diagram for recording intestinal vagus nerve activities during optogenetic activation of ChR2-containing DMV^{ACh} terminals (pulse: 2 ms; frequency: 50 Hz; duration: 100 ms). **h**, Representative images showing co-localization of the mCherry and TUBB3 antibody signals. Scale bar, 50 μm. **i**,**j**, Representative traces (**i**) and summary data (**j**) for intestinal vagus nerve electronic activities during optogenetic activation of DMV^{Ach} neuronal fibers (n = 6, P = 0.2437 for mCherry, P = 0.0034 for ChR2). Significance was assessed by two-tailed unpaired

Student's *t*-test (**c**) and two-tailed paired Student's *t*-test (**j**). The data are presented as the mean ± SEMs. $^{**}P < 0.01$; n.s., not significant. See also Supplementary Table S1. Source data are provided as a Source Data file.

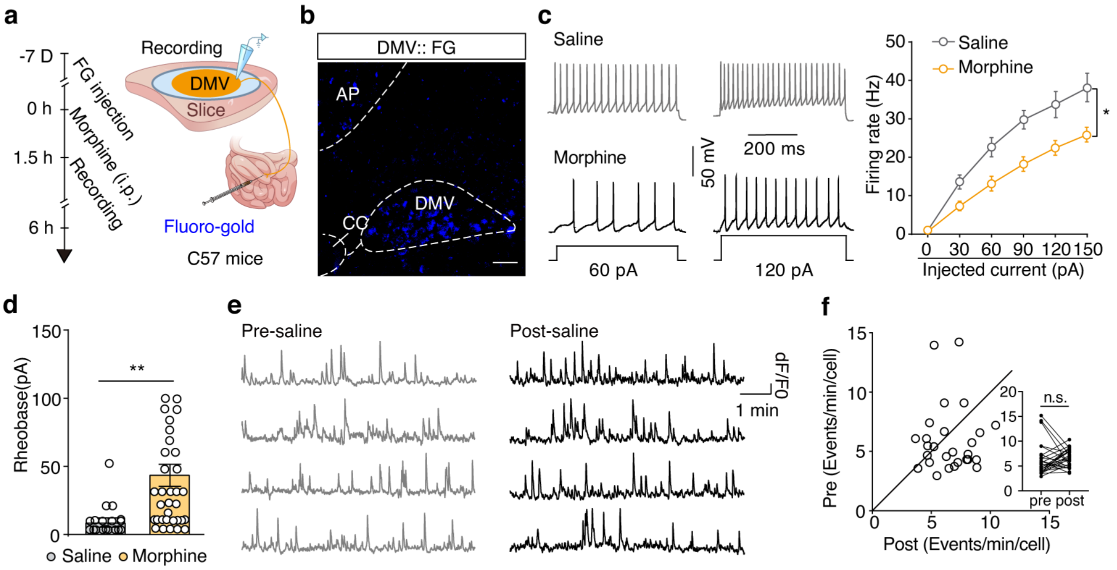

Supplementary Fig. 3 | Morphine reduces DMV^{Ach} neuronal activity. a, Schematic for FG injection in the small intestine of morphine-treated mice and the whole-cell recording configuration in acute slices. **b**, A representative image of FG expression in the DMV. Scale bar, 50 μm. **c**, Sample traces (left) and summary data (right) for evoked action potentials recorded from the small intestine-projecting DMV neurons (n = 18 for saline, n = 35 for morphine, $F_{1,51}$ = 5.415, P = 0.0240). **d**, Summary data for the rheobase from the small intestine-projecting DMV neurons (n = 18 for saline, n = 35 for morphine, U = 132.5, P = 0.0003). **e**,**f**, Sample traces (**e**) and summary data (**f**) for calcium transients of GCaMP6m-expressing DMV neurons before and after saline injection (n = 28 cells per group, P = 0.2304). Significance was assessed by two-way repeated-measures ANOVA with post hoc comparison between groups (**c**), Mann-Whitney U test (**d**), and two-tailed paired Student's t-test (**f**). The data are presented as the mean ± SEMs. *P < 0.05, **P < 0.01; n.s., not significant. See also Supplementary Table S1. Source data are provided as a Source Data file.

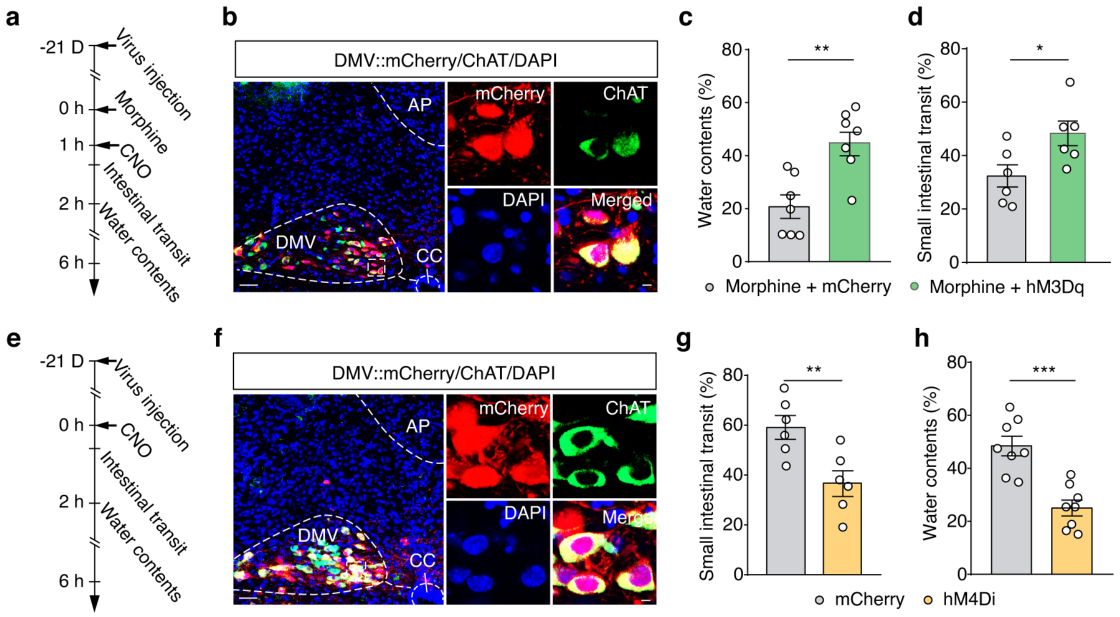

Supplementary Fig. 4 | Chemogenetic modulation of DMV^{Ach} neurons affects small intestinal function. a, Schematic diagram for AAV-DIO-hM3Dq-mCherry virus injection into the DMV and AAV2/Retro-hSyn-Cre virus injection into the small intestine of C57 mice. **b**, Representative images showing co-localization of mCherry^{+} neurons with a ChAT-specific antibody in the DMV. Scale bars, 50 µm (overview) and 5 µm (zoom). **c**,**d**, Summary data for the water contents of feces (**c**) and gastrointestinal transit rate (**d**) in the morphine-treated mice from mCherry and hM3Dq groups (Water contents: n = 7, $P = 0.0033$; small intestinal transit: n = 6, $P = 0.0187$). **e-h**, As indicated in panels **a-d**, but for chemogenetic inhibition of the small intestine-projecting DMV neurons in naïve mice. Scale bars, 50 µm (overview) and 5 µm (zoom) (Small intestinal transit: n = 6, $P = 0.0097$; water contents: n = 8, $P = 0.0001$). Significance was assessed by two-tailed unpaired Student's *t*-test (**c**, **d**, **g** and **h**). The data are presented as the mean ± SEMs. $*P < 0.05$, $**P < 0.01$, $***P < 0.001$; n.s., not significant. See also Supplementary Table S1. Source data are provided as a Source Data file.

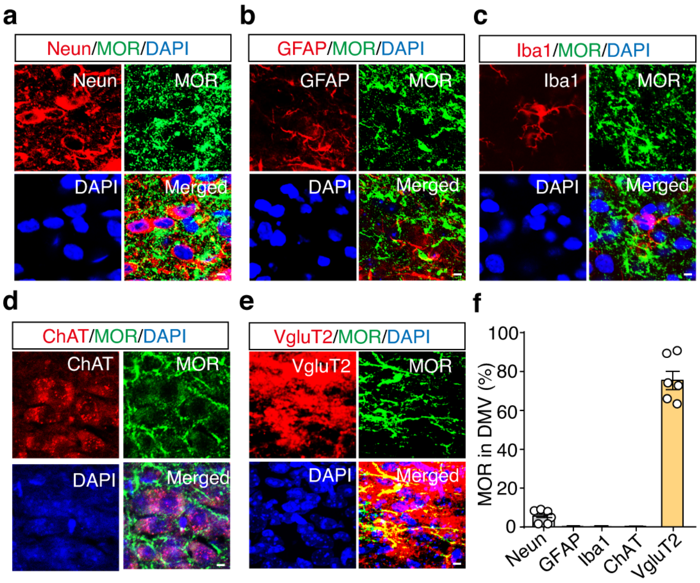

Supplementary Fig. 5 | Cell type identifications of MOR-expressing neurons in the DMV. a-f, Representative images (**a**-**e**) and summary data (**f**) for the co-localization of MOR with the neuronal nuclei (NeuN, a marker for neuron), glial fibrillary acidic protein (GFAP, a maker for astrocyte), ionized calcium-binding adapter molecule 1 (Iba1, a marker for microglia), or a ChAT-specific antibody, or VgluT2^{+}signals in the DMV of *VgluT2-Ai* transgenic mice, respectively. Scale bars, 5 µm. The data are presented as the mean ± SEMs. Source data are provided as a Source Data file.

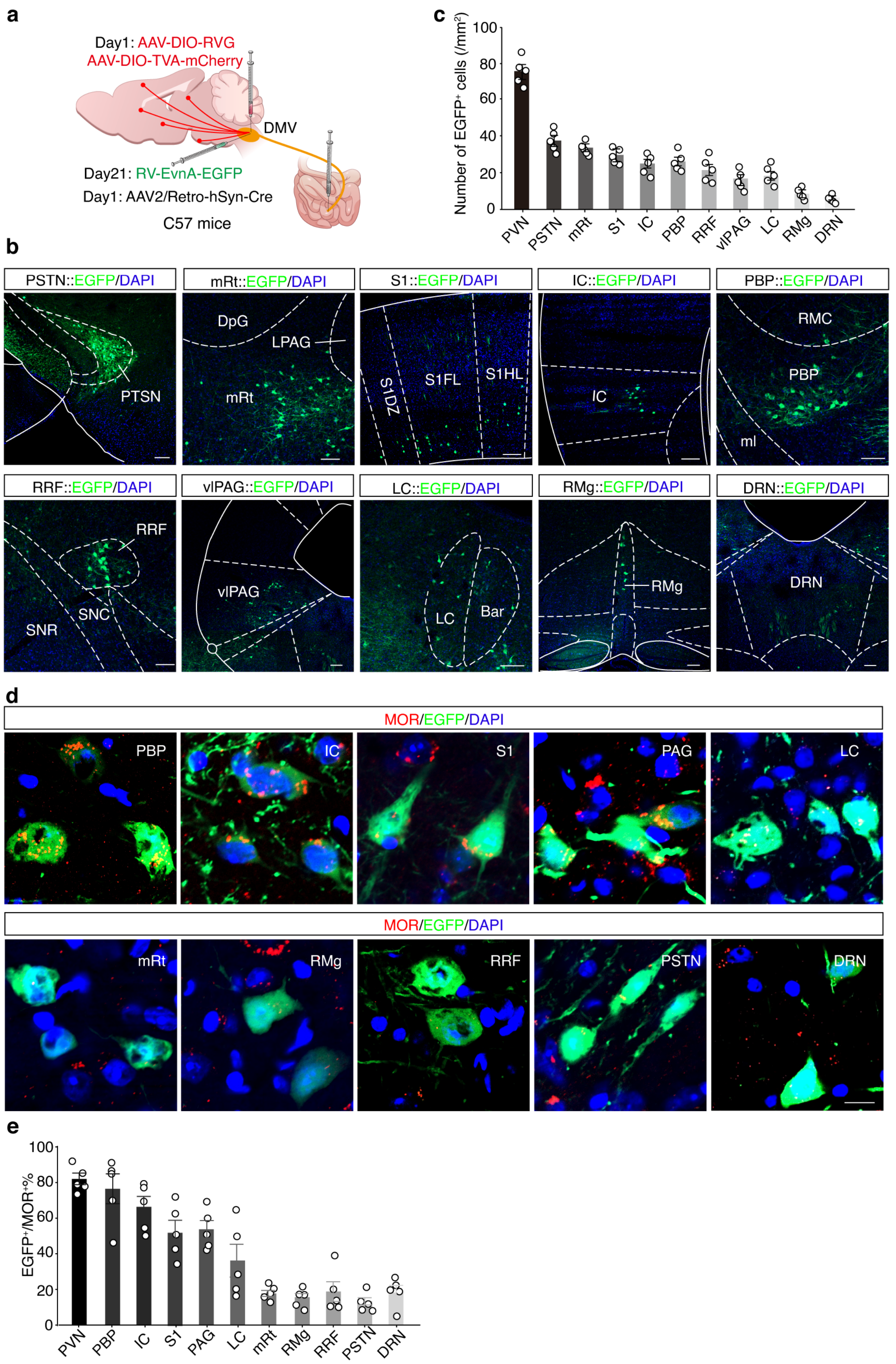

Supplementary Fig. 6 | Retrograde tracing of the PVN^{Glu}→DMV^{Ach}→small intestine circuit and MOR expression. a, Schematic diagram for the Cre-dependent

retrograde trans-monosynaptic rabies virus (RV) tracing strategy. **b**, Representative images of EGFP$^+$ neurons in multiple brain regions, including the paraventricular nucleus of hypothalamus (PVN), the parasubthalamic nucleus (PSTN), the mesencephalic reticular formation (mRt), the somatosensory cortex(S1), the insula cortex (IC), the parabrachial pigmented nucleus (PBP), the retrorubral field (RRF), the ventrolateral periaqueductal gray (vlPAG), the locus coeruleus (LC), the raphe magnus nucleus (RMg), and the dorsal raphe nucleus (DRN). Scale bars, 100 μm. **c**, Summary data showing the distribution of EGFP$^+$ neurons across the above brain regions. **d**, Representative images showing MOR expression in EGFP-labeled neurons across multiple brain regions, identified using a Cre-dependent retrograde trans-monosynaptic rabies virus (RV) tracing strategy. Scale bars, 10 μm. **e**, Summary data showing the distribution of MOR-positive EGFP-labeled neurons across these brain regions. The data are presented as the mean ± SEMs. Source data are provided as a Source Data file.

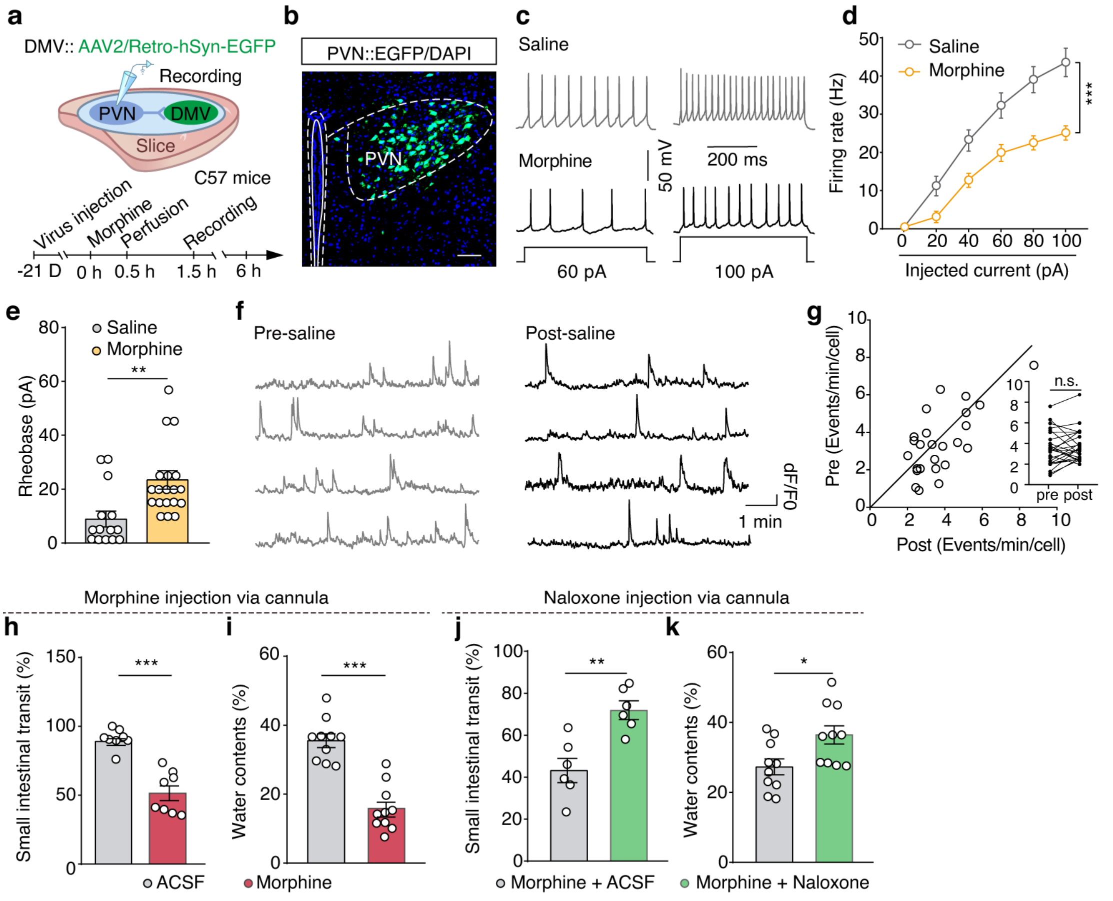

Supplementary Fig. 7 | Morphine reduces DMV-projecting PVN neuronal activity. a, Schematic diagram for virus injection in morphine-treated mice and the whole-cell recording configuration in acute slices. **b**, Representative image of viral expression in the PVN. Scale bar, 50 μm. **c**,**d**, Sample traces (**c**) and summary data (**d**) for evoked action potentials recorded from the DMV-projecting PVN neurons (n = 14 for saline, n = 19 for morphine, $F_{1,31}$ = 16.07, P = 0.0004). **e**, Summary data for the Rheobase from the DMV-projecting PVN neurons (n = 14 for saline, n = 19 for morphine, U = 49.50, P = 0.0016). **f**,**g**, Sample traces (**f**) and summary data (**g**) for calcium transients of GCaMP6m-expressing PVN neurons before and after saline injection (n = 25 cells per group, P = 0.3886). **h**,**i**, Summary data for the small intestinal transit rate (**h**) and fecal water content (**i**) in mice after bilaterally administrated morphine into the PVN via cannulas (Small intestinal transit: n = 8, P < 0.0001; water contents: n = 10, P < 0.0001). **j**,**k**, Summary data for the small intestinal transit rate (**j**) and fecal water content (**k**) in mice after bilaterally administrated naloxone into the PVN via cannulas (Small intestinal transit: n = 6, P = 0.0029; water contents: n = 10, P

= 0.0209). Significance was assessed by two-way repeated-measures ANOVA with post hoc comparison between groups (**d**), Mann-Whitney U test (**e**), two-tailed paired Student's *t*-test (**g**) and two-tailed unpaired Student's *t*-test (**h-k**). The data are presented as the mean ± SEMs. *$P < 0.05$, **$P < 0.01$, ***$P < 0.001$; n.s., not significant. See also Supplementary Table S1. Source data are provided as a Source Data file.

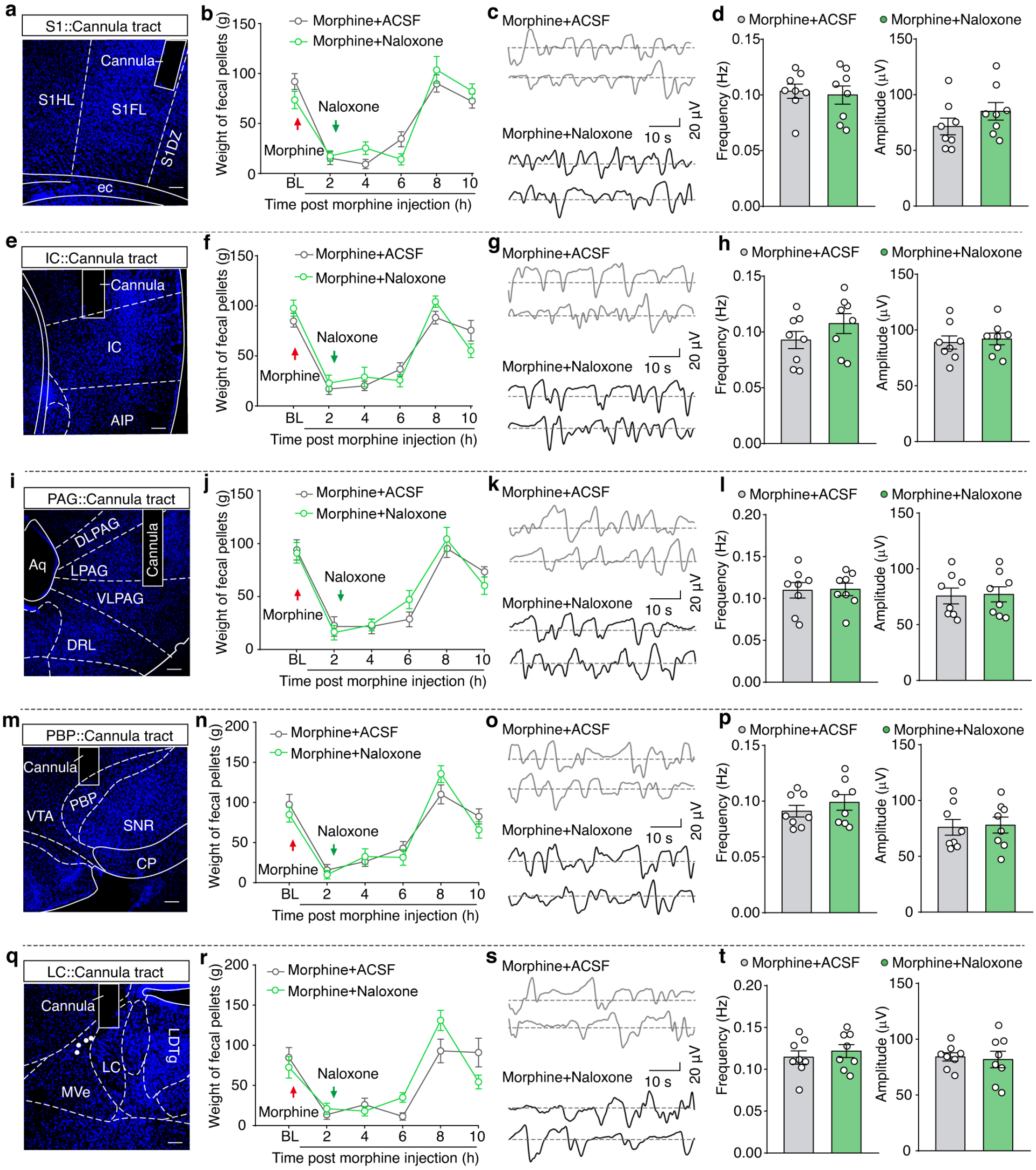

Supplementary Fig. 8 | Administering MOR antagonist in S1, IC, PAG, PBP or LC does not affect morphine-induced constipation. a-d, Representative images (**a**), summary data for fecal weight (**b**), sample traces from strain gauge (**c**) and summary data of frequency and amplitude values (**d**) from S1, Scale bar, 100 μm (n = 8, $F_{1,14}$ = 0.05293, P = 0.8214 for weight; n = 8, P = 0.7449 for frequency, P = 0.2324 for amplitude). **e-h**, As indicated in panels **a-d**, but for the region of IC (n = 8, $F_{1,14}$ = 0.3791, P = 0.5480 for weight; n = 8, P = 0.2318 for frequency, P =0.6870 for amplitude). **i-l**, As indicated in panels **a-d**, but for the region of PAG (n = 8, $F_{1,14}$ = 0.05882, P = 0.8119 for weight; n = 8, P = 0.9303 for frequency, P = 0.8820 for

amplitude). **m-p**, As indicated in panels **a**-**d**, but for the region of PBP (n = 8, $F_{1,14}$ = 0.4154, P = 0.5297 for weight; n = 8, P = 0.3870 for frequency, P = 0.8465 for amplitude). **q-t**, As indicated in panels **a**-**d**, but for the region of LC (n = 8, $F_{1,14}$ = 0.1911, P = 0.6687 for weight; n = 8, P = 0.5058 for frequency, P = 0.7852 for amplitude). Significance was assessed by two-way repeated-measures ANOVA with post hoc comparison between groups (**b**, **f**, **j**, **n** and **r**) and two-tailed unpaired Student's *t*-test (**d**, **h**, **l**, **p** and **t**). The data are presented as the mean ± SEMs. See also Supplementary Table S1. Source data are provided as a Source Data file.

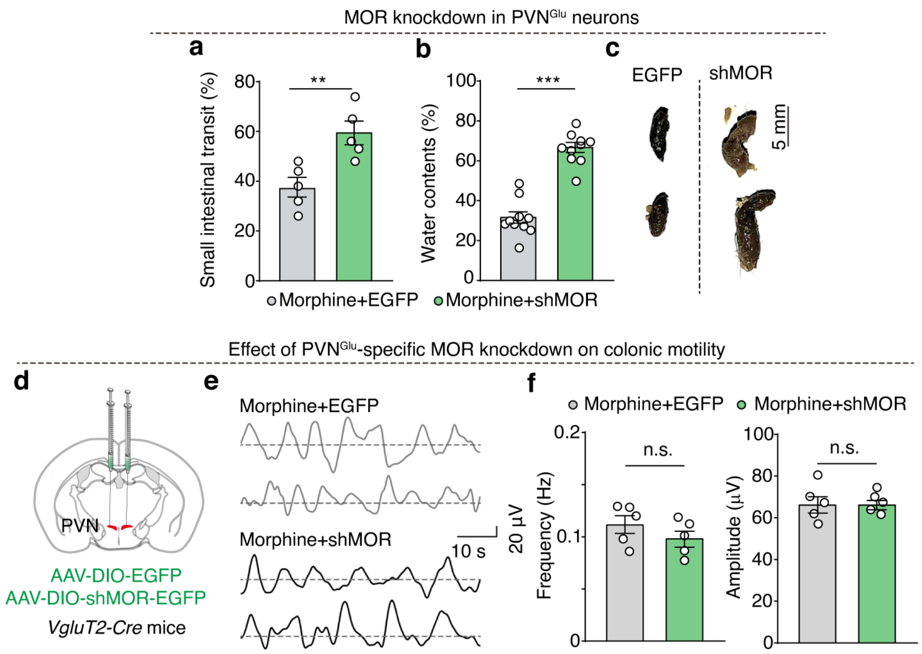

Supplementary Fig. 9 | MOR knockdown in PVN^{Glu} neurons alleviates morphine-induced inhibition of small intestinal motility, but not colonic motility. a,**b**, In mice with morphine-induced constipation, MOR knockdown in PVN^{Glu} neurons increased the small intestinal transit (**a**, n = 5, P = 0.0080) and water contents (**b**, n = 10, P < 0.0001). **c**, Representative image of fecal size in mice with PVN^{Glu}-specific MOR knockdown. **d**, Schematic diagram for MOR knockdown in PVN^{Glu} neurons of morphine-treated mice. **e**,**f**, Sample traces (**e**) and summary data (**f**) showing the frequency and amplitude values for colon motility in the PVN^{Glu} neurons-specific MOR knockdown mice after morphine injection (n = 5, P = 0.2910 for frequency, P = 0.9904 for amplitude). Significance was assessed by two-tailed unpaired Student's *t*-test (**a**, **b** and **f**). The data are presented as the mean ± SEMs. **P < 0.01, ***P < 0.001, n.s., not significant. See also Supplementary Table S1. Source data are provided as a Source Data file.

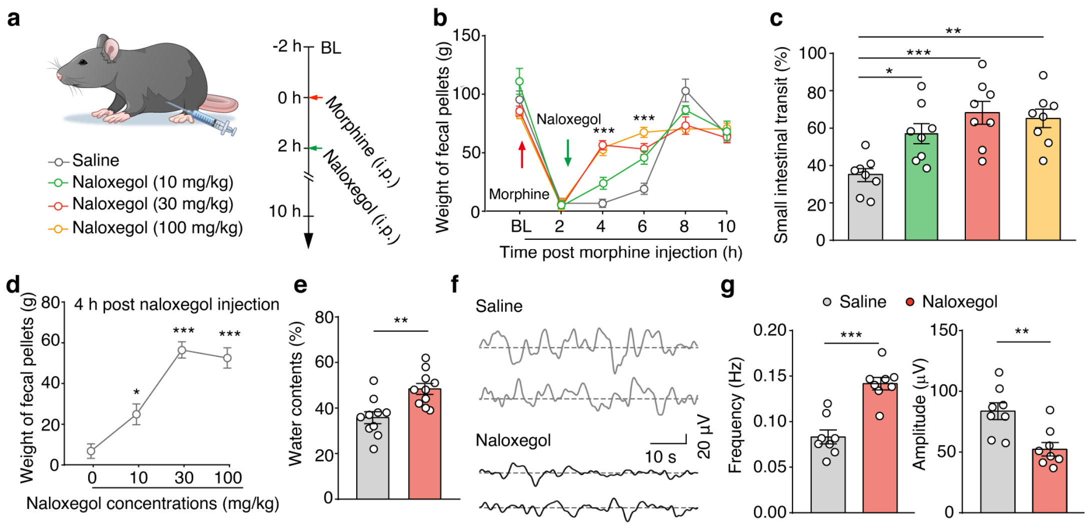

Supplementary Fig. 10 | Naloxegol relieves morphine-induced constipation. a, Schematic diagram for intraperitoneal injection of naloxegol at different concentrations. **b**,**c**, Summary data for fecal weight (**b**) and small intestinal transit rate (**c**) of morphine-treated mice at different naloxegol concentrations (Fecal weight: n = 10, $F_{3,36}$ = 3.899, P < 0.0001 for 4 h; P < 0.0001 for 6 h; Transit rate: n = 8, $F_{3,28}$ = 8.797, P = 0.0003). **d**, Dose-dependent reduction in fecal weight at different naloxegol concentrations (n = 10, $F_{3,36}$ = 28.08, P < 0.0001). **e**, Summary data for fecal water contents following intraperitoneal injection of morphine and naloxegol (n = 10, P = 0.0025). **f**,**g**, Sample traces (**f**) and summary data (**g**) showing the frequency and amplitude values for small intestinal motility following intraperitoneal injection of morphine and naloxegol (n = 8; Frequency: P < 0.0001; Amplitude: P = 0.0041). Significance was assessed by two-way repeated-measures ANOVA with post hoc comparison between groups (**b**), one-way ANOVA with post hoc Bonferroni's test between groups (**c**, **d**) and two-tailed unpaired Student's *t*-test (**e**, **g**). The data are presented as the mean ± SEMs. *P < 0.05, **P < 0.01, ***P < 0.001; n.s., not significant. See also Supplementary Table S1. Source data are provided as a Source Data file.

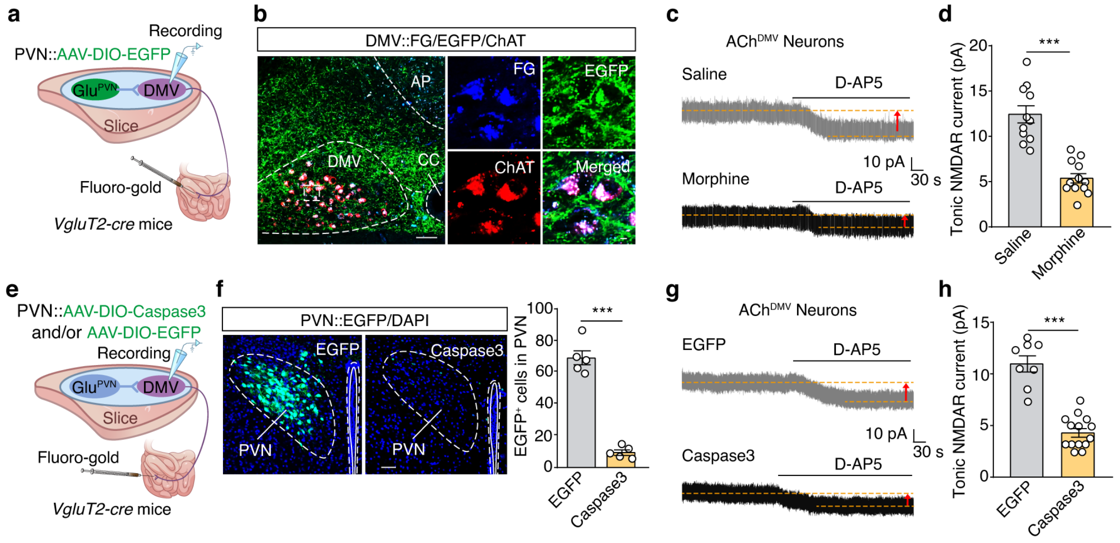

Supplementary Fig. 11 | Morphine suppresses tonic NMDA receptor-mediated currents in the DMV^{Ach} neurons. a, Schematic diagram for FG and virus injections in morphine-treated *VgluT2-Cre* mice, and the whole-cell recording configuration in acute slices. **b**, Representative images of PVN neuronal terminals surrounding the small intestine-projecting DMV neurons. Scale bar, 50 µm. **c**,**d**, Representative traces (**c**) and summary data (**d**) for the tonic currents recorded from the small intestine-projecting DMV neurons in saline and morphine groups (n = 11 for Saline, n = 12 for Morphine, $P < 0.0001$). **e-h**, As indicated in panels **a-d**, but for the ablation of PVN^{Glu} neurons by infusing AAV-DIO-taCaspase3 virus into the PVN of *VgluT2-Cre* mice (EGFP^{+} cell: n = 5 mice, $P < 0.0001$; Tonic current: n = 8 for EGFP, n = 14 for Caspase3, $P < 0.0001$). Significance was assessed by two-tailed unpaired Student's *t*-test (**d**, **f** and **h**). The data are presented as the mean ± SEMs. ***$P < 0.001$. See also Supplementary Table S1. Source data are provided as a Source Data file.

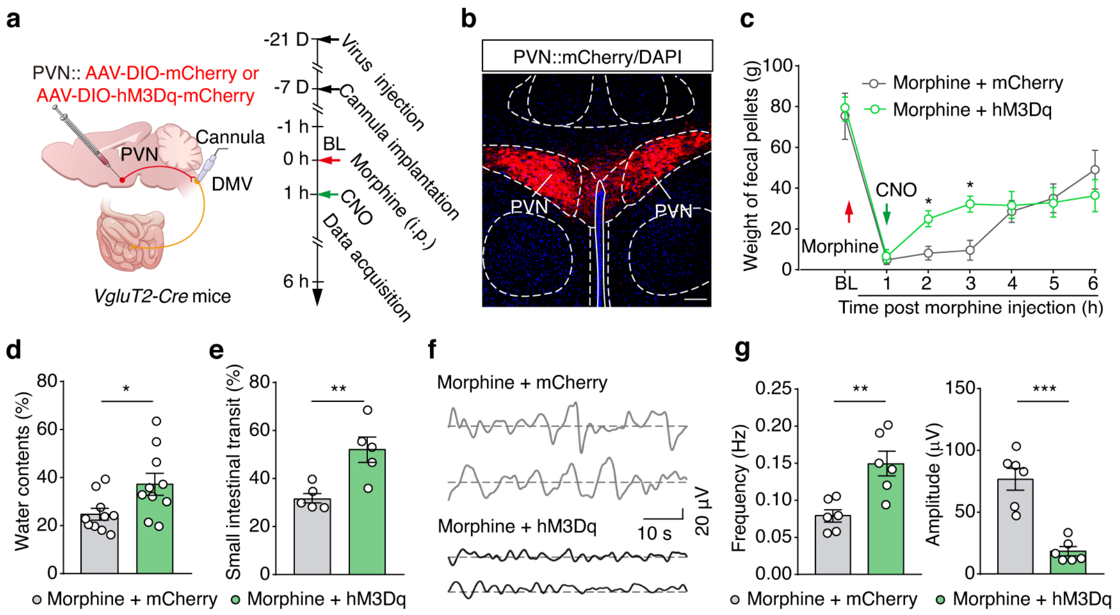

Supplementary Fig. 12 | Chemogenetic activation of DMV-projecting PVN^{Glu} neurons alleviates morphine-induced constipation. **a**, Schematic diagram for chemogenetic activation of DMV-projecting PVN^{Glu} neurons and cannula implantation in the DMV of *VgluT2-Cre* mice. **b**, Representative images for mCherry expression in the PVN. Scale bar, 100 μm. **c-e**, Summary data for fecal weight (**c**), fecal water content (**d**), and small intestinal transit rate (**e**) in the mCherry-expressing controls and hM3Dq-expressing mice (n = 10, $F_{1,18}$ = 1.069, P = 0.0356 for 2 h, P = 0.0220 for 3 h; water contents: n = 10, P = 0.0226; small intestinal transit: n = 5, P = 0.0072). **f**,**g**, Sample traces (**f**) and summary data (**g**) showing the frequency and amplitude values for small intestinal motility in the mCherry-expressing controls and hM3Dq-expressing mice (n = 6, P = 0.0035 for frequency, P = 0.0001 for amplitude). Significance was assessed by two-way repeated-measures ANOVA with post hoc comparison between groups (**c**) and two-tailed unpaired Student's *t*-test (**d**, **e** and **g**). The data are presented as the mean ± SEMs. $^*P < 0.05$, $^{**}P < 0.01$, $^{***}P < 0.001$; n.s., not significant. See also Supplementary Table S1. Source data are provided as a Source Data file.

Table S1. Statistical analyses related to figures 1-7 and Supplementary figures S1-S12.

Figure	Conditions (sample size)		Analysis	*P* value	t or F or U value
Fig. 1b	Saline (10)	Morphine (10)	Two-way RM ANOVA with Bonferroni post hoc analysis	$P < 0.0001$	$F_{(1, 18)} = 28.75$
	BL			$P > 0.9999$	
	2 h			$P < 0.0001$	
	4 h			$P = 0.0392$	
	6 h			$P = 0.0193$	
	8 h			$P > 0.9999$	
	10 h			$P > 0.9999$	
Fig. 1c	0mg/kg (10), 3mg/kg (10), 6mg/kg (10), 10mg/kg (10)		Ordinary one-way ANOVA with Bonferroni post hoc analysis	$P = 0.0002$	$F_{(3, 36)} = 8.310$
	0 mg/kg	3 mg/kg		$P = 0.2207$	
	0 mg/kg	6 mg/kg		$P = 0.0225$	
	0 mg/kg	10 mg/kg		$P < 0.0001$	
Fig. 1d	Saline (8)	Morphine (8)	Two-way RM ANOVA with Bonferroni post hoc analysis	$P = 0.0001$	$F_{(1, 14)} = 27.25$
	BL			$P > 0.9999$	
	2 h			$P = 0.0143$	
	4 h			$P = 0.0013$	

	6 h			$P = 0.0005$	
	8 h			$P > 0.9999$	
	10 h			$P > 0.9999$	
Fig. 1e	Control (7), Morphine 3 mg/kg (7), Morphine 6 mg/kg (7), Morphine 10 mg/kg (7)		Ordinary one-way ANOVA with Bonferroni post hoc analysis	$P < 0.0001$	$F_{(3, 24)} = 22.61$
	Control	Morphine 3 mg/kg		$P = 0.0173$	
	Control	Morphine 6 mg/kg		$P = 0.0001$	
	Control	Morphine 10 mg/kg		$P < 0.0001$	
Fig. 1h	Frequency: Saline (8)	Frequency: Morphine (8)	Two-tailed unpaired t-test	$P < 0.0001$	$t_{(14)} = 10.81$
	Amplitude: Saline (8)	Amplitude: Morphine (8)	Two-tailed unpaired t-test	$P < 0.0001$	$t_{(14)} = 10.64$
Fig. 1j	Frequency: Saline (5)	Frequency: Morphine (5)	Two-tailed unpaired t-test	$P = 0.0144$	$t_{(8)} = 3.113$
	Amplitude: Saline (5)	Amplitude: Morphine (5)	Two-tailed unpaired t-test	$P = 0.0019$	$t_{(8)} = 4.555$
Fig. 2l	Frequency: Saline (6)	Frequency: Morphine (6)	Two-tailed unpaired t-test	$P = 0.0002$	$t_{(10)} = 5.603$
	Amplitude: Saline (6)	Amplitude: Morphine (6)	Two-tailed unpaired t-test	$P < 0.0001$	$t_{(10)} = 9.487$
Fig. 3d	Saline (28)	Morphine (28)	Two-tailed paired t-test	$P < 0.0001$	$t_{(27)} = 8.092$
Fig. 3f	mCherry (7)	hM3Dq (7)	Two-way RM ANOVA with Bonferroni post hoc analysis	$P = 0.4622$	$F_{(1, 12)} = 0.5770$
	BL			$P > 0.9999$	

	1 h			$P > 0.9999$	
	2 h			$P = 0.0341$	
	3 h			$P = 0.3889$	
	4 h			$P > 0.9999$	
	5 h			$P > 0.9999$	
	6 h			$P > 0.9999$	
Fig. 3h	Frequency: mCherry (5)	Frequency: hM3Dq (5)	Two-tailed unpaired t-test	$P = 0.0067$	$t_{(8)} = 3.631$
	Amplitude: mCherry (5)	Amplitude: hM3Dq (5)	Two-tailed unpaired t-test	$P = 0.0005$	$t_{(8)} = 5.660$
Fig. 3j	mCherry (7)	hM4Di (7)	Two-way RM ANOVA with Bonferroni post hoc analysis	$P < 0.0001$	$F_{(1, 12)} = 37.11$
	BL			$P > 0.9999$	
	1 h			$P = 0.0058$	
	2 h			$P = 0.0427$	
	3 h			$P = 0.0621$	
	4 h			$P > 0.9999$	
	5 h			$P > 0.9999$	
	6 h			$P = 0.5561$	
Fig. 3l	Frequency: mCherry (5)	Frequency: hM4Di (5)	Two-tailed unpaired t-test	$P = 0.0002$	$t_{(8)} = 6.449$
	Amplitude: mCherry (5)	Amplitude: hM4Di (5)	Two-tailed unpaired t-test	$P < 0.0001$	$t_{(8)} = 8.809$
Fig. 4m	ACSF (5)	DNQX (5)	Two-tailed unpaired t-test	$P < 0.0001$	$t_{(8)} = 14.09$
Fig. 5f	Saline (25)	Morphine (25)	Two-tailed paired t-test	$P = 0.0031$	$t_{(24)} = 3.284$

Fig. 5h	ACSF (10)	Morphine (10)	Two-way RM ANOVA with Bonferroni post hoc analysis	$P = 0.0007$	$F_{(1, 18)} = 16.89$
	BL			$P > 0.9999$	
	2 h			$P < 0.0001$	
	4 h			$P < 0.0001$	
	6 h			$P > 0.9999$	
	8 h			$P = 0.023$	
	10 h			$P > 0.9999$	
Fig. 5j	Frequency: ACSF (10)	Frequency: Morphine (10)	Mann-Whitney U test	$P = 0.0021$	U=11
	Amplitude: ACSF (10)	Amplitude: Morphine (10)	Two-tailed unpaired t-test	$P < 0.0001$	$t_{(18)} = 11.36$
Fig. 5l	ACSF (10)	Morphine (10)	Two-way RM ANOVA with Bonferroni post hoc analysis	$P = 0.0292$	$F_{(1, 18)} = 5.612$
	BL			$P > 0.9999$	
	2 h			$P > 0.9999$	
	4 h			$P = 0.0166$	
	6 h			$P = 0.2949$	
	8 h			$P > 0.9999$	
	10 h			$P > 0.9999$	
Fig. 5n	Frequency: ACSF (10)	Frequency: Naloxone (10)	Two-tailed unpaired t-test	$P < 0.0001$	$t_{(18)} = 10.89$
	Amplitude: ACSF (10)	Amplitude: Naloxone (10)	Mann-Whitney U test	$P < 0.0001$	U = 0

Fig. 6c	EGFP (4)	shMOR (4)	Two-tailed unpaired t-test	$P = 0.0003$	$t_{(6)} = 7.641$
Fig. 6e	Morphine+EGFP (11)	Morphien+shMOR (10)	Two-way RM ANOVA with Bonferroni post hoc analysis	$P = 0.0001$	$F_{(1, 19)} = 23.10$
Fig. 6f	Morphine+EGFP (10)	Morphien+shMOR (10)	Two-way RM ANOVA with Bonferroni post hoc analysis	$P = 0.0429$	$F_{(1, 18)} = 4.748$
	BL			$P = 0.1771$	
	2 h			$P = 0.0059$	
	4 h			$P = 0.0414$	
	6 h			$P = 0.0381$	
	8 h			$P = 0.2078$	
	10 h			$P > 0.9999$	
Fig. 6h	Frequency: Morphine+EGFP (6)	Frequency: Morphine+shMOR (6)	Two-tailed unpaired t-test	$P = 0.0189$	$t_{(10)} = 2.798$
	Amplitude: Morphine+EGFP (6)	Amplitude: Morphine+shMOR (6)	Two-tailed unpaired t-test	$P < 0.0001$	$t_{(10)} = 7.219$
Fig. 6j	EGFP+Naloxegol (8)	shMOR+Naloxegol (8)	Two-way RM ANOVA with Bonferroni post hoc analysis	$P = 0.1034$	$F_{(1, 14)} = 3.035$
	BL			$P = 0.2489$	
	2 h			$P = 0.0390$	
	4 h			$P = 0.0762$	
	6 h			$P = 0.0071$	

	8 h			$P = 0.0199$	
	10 h			$P > 0.9999$	
Fig. 6l	Frequency: EGFP+Naloxegol (6)	Frequency: shMOR+Naloxegol (6)	Two-tailed unpaired t-test	$P = 0.0421$	$t_{(10)} = 2.330$
	Amplitude: EGFP+Naloxegol (6)	Amplitude: shMOR+Naloxegol (6)	Two-tailed unpaired t-test	$P = 0.0115$	$t_{(10)} = 3.090$
Fig. 7c	EGFP (10)	hM4Di (10)	Two-way RM ANOVA with Bonferroni post hoc analysis	$P = 0.1744$	$F_{(1, 18)} = 1.999$
	BL			$P > 0.9999$	
	1 h			$P < 0.0001$	
	2 h			$P = 0.0004$	
	3 h			$P = 0.0291$	
	4 h			$P = 0.0714$	
	5 h			$P = 0.9529$	
	6 h			$P > 0.9999$	
Fig. 7d	EGFP (10)	hM4Di (10)	Two-tailed unpaired t-test	$P < 0.0001$	$t_{(18)} = 5.428$
Fig. 7e	EGFP (5)	hM4Di (5)	Two-tailed unpaired t-test	$P = 0.0009$	$t_{(8)} = 5.095$
Fig. 7g	Frequency: EGFP (6)	Frequency: hM4Di (6)	Mann-Whitney U test	$P = 0.0022$	U = 0
	Amplitude: EGFP (6)	Amplitude: hM4Di (6)	Two-tailed unpaired t-test	$P < 0.0001$	$t_{(10)} = 10.27$
Fig. 7j	Morphine+mCherry (10)	Morphine+ChR2 (10)	Two-way RM ANOVA with Bonferroni post hoc analysis	$P = 0.7309$	$F_{(1, 18)} = 0.1220$

	BL			$P = 0.8459$	
	2 h			$P > 0.9999$	
	4 h			$P < 0.0001$	
	6 h			$P = 0.8811$	
	8h			$P = 0.3319$	
	10 h			$P > 0.9999$	
Fig. 7k	Morphine+mCherry (10)	Morphine+ChR2 (10)	Two-tailed unpaired t-test	$P = 0.0017$	$t_{(18)} = 3.696$
Fig. 7l	Morphine+mCherry (8)	Morphine+ChR2 (8)	Two-tailed unpaired t-test	$P = 0.0360$	$t_{(14)} = 2.319$
Fig. 7n	Frequency: Morphine+mCherry (10)	Frequency: Morphine+ChR2 (10)	Two-tailed unpaired t-test	$P = 0.0003$	$t_{(18)} = 4.532$
	Amplitue: Morphine+mCherry (10)	Amplitude: Morphine+ChR2 (10)	Two-tailed unpaired t-test	$P < 0.0001$	$t_{(18)} = 4.972$
Supplementary Fig. 1a	Saline (10)	Morphine (3 mg/kg) (10)	Two-way RM ANOVA with Bonferroni post hoc analysis	$P = 0.0649$	$F_{(1, 18)} = 3.867$
	BL			$P > 0.9999$	
	2 h			$P = 0.4460$	
	4 h			$P = 0.2082$	
	6 h			$P = 0.0593$	
	8 h			$P = 0.0573$	
	10 h			$P > 0.9999$	
Supplementary Fig. 1a	Saline (10)	Morphine (6 mg/kg) (10)	Two-way RM ANOVA with Bonferroni post hoc analysis	$P = 0.0469$	$F_{(1, 18)} = 4.554$
	BL			$P > 0.9999$	
	2 h			$P = 0.026$	

	4 h			$P = 0.0409$	
	6 h			$P = 0.3033$	
	8 h			$P > 0.9999$	
	10 h			$P > 0.9999$	
Supplementary Fig. 1b	Saline (8)	Morphine (8)	Two-way RM ANOVA with Bonferroni post hoc analysis	$P = 0.9245$	$F_{(1, 14)} = 0.009299$
	BL			$P > 0.9999$	
	2 h			$P = 0.1002$	
	4 h			$P > 0.9999$	
	6 h			$P = 0.4286$	
	8 h			$P > 0.9999$	
	10 h			$P > 0.9999$	
	Saline (8)	Morphine (8)	Mann-Whitney U test	$P = 0.9591$	U= 31
Supplementary Fig. 1c	Saline (8)	Morphine (8)	Two-way RM ANOVA with Bonferroni post hoc analysis	$P = 0.8408$	$F_{(1, 14)} = 0.04189$
	BL			$P > 0.9999$	
	2 h			$P = 0.8130$	
	4 h			$P > 0.9999$	
	6 h			$P = 0.3439$	
	8 h			$P > 0.9999$	
	10 h			$P > 0.9999$	
	Saline (8)	Morphine (8)	Mann-Whitney U test	$P = 0.2786$	U = 21
Supplementary Fig. 2c	Left Side (5)	Right Side (5)	Two-tailed unpaired t-test	$P = 0.4991$	$F_{(1, 8)} = 0.5010$

Supplementary Fig. 2j	mCherry: Pre (6)	mCherry: Light (6)	Two-tailed paired t-test	$P = 0.2437$	$t_{(5)} = 1.321$
	ChR2: Pre (6)	ChR2: Light (6)	Two-tailed paired t-test	$P = 0.0034$	$t_{(5)} = 0.0034$
Supplementary Fig. 3c	Saline (18)	Morphine (35)	Two-way RM ANOVA with Bonferroni post hoc analysis	$P = 0.0240$	$F_{(1, 51)} = 5.415$
Supplementary Fig. 3d	Saline (18)	Morphine (35)	Mann-Whitney U test	$P = 0.0003$	U =132.5
Supplementary Fig. 3f	Pre-saline (28)	Post-saline (28)	Two-tailed paired t-test	$P = 0.2304$	$t_{(27)} = 1.227$
Supplementary Fig. 4c	Morphine+mCherry(7)	Morphine+hM3Dq (7)	Two-tailed unpaired t-test	$P = 0.0033$	$t_{(12)} = 3.646$
Supplementary Fig. 4d	Morphine+mCherry(6)	Morphine+hM3Dq (6)	Two-tailed unpaired t-test	$P = 0.0187$	$t_{(10)} = 2.804$
Supplementary Fig. 4g	mCherry(6)	hM4Di (6)	Two-tailed unpaired t-test	$P = 0.0097$	$t_{(10)} = 3.184$
Supplementary Fig. 4h	mCherry(8)	hM4Di (8)	Two-tailed unpaired t-test	$P = 0.0001$	$t_{(14)} = 5.148$
Supplementary Fig. 7d	Saline (14)	Morphine (19)	Two-way RM ANOVA with Bonferroni post hoc analysis	$P = 0.0004$	$F_{(1, 31)} = 16.07$
Supplementary Fig. 7e	Saline (14)	Morphine (19)	Mann-Whitney U test	$P = 0.0016$	U = 49.50
Supplementary Fig. 7g	Pre-saline (25)	Post-saline (25)	Two-tailed paired t-test	$P = 0.3886$	$t_{(14)} = 0.8780$
Supplementary Fig.	ACSF (8)	Morphine (8)	Two-tailed unpaired t-	$P < 0.0001$	$t_{(14)} = 6.459$

7h			test		
Supplementary Fig. 7i	ACSF (10)	Morphine (10)	Two-tailed unpaired t-test	$P < 0.0001$	$t_{(18)} = 6.806$
Supplementary Fig. 7j	ACSF (6)	Naloxone (6)	Two-tailed unpaired t-test	$P = 0.0029$	$t_{(10)} = 3.917$
Supplementary Fig. 7k	ACSF (10)	Naloxone (10)	Two-tailed unpaired t-test	$P = 0.0209$	$t_{(18)} = 2.531$
Supplementary Fig. 8b	Morphine+ACSF(8)	Morphine+Naloxone (8)	Two-way RM ANOVA with Bonferroni post hoc analysis	$P = 0.8214$	$F_{(1, 14)} = 0.05293$
	BL			$P = 0.5700$	
	2 h			$P > 0.9999$	
	4 h			$P = 0.8899$	
	6 h			$P = 0.4074$	
	8 h			$P > 0.9999$	
	10 h			$P > 0.9999$	
Supplementary Fig. 8d	Frequency: Morphine+ACSF (8)	Frequency: Morphine+Naloxone (8)	Two-tailed unpaired t-test	$P = 0.7449$	$t_{(14)} = 0.3319$
	Amplitude: Morphine+ACSF (8)	Amplitude: Morphine+Naloxone (8)	Two-tailed unpaired t-test	$P = 0.2324$	$t_{(14)} = 1.248$
Supplementary Fig. 8f	Morphine+ACSF(8)	Morphine+Naloxone (8)	Two-way RM ANOVA with Bonferroni post hoc analysis	$P = 0.5480$	$F_{(1, 14)} = 0.3791$
	BL			$P > 0.9999$	
	2 h			$P > 0.9999$	
	4 h			$P > 0.9999$	
	6 h			$P > 0.9999$	
	8 h			$P = 0.4660$	
	10 h			$P = 0.8073$	

Supplementary Fig. 8h	Frequency: Morphine+ACSF (8)	Frequency: Morphine+Naloxone (8)	Two-tailed unpaired t-test	$P = 0.2318$	$t_{(14)} = 1.250$
	Amplitude: Morphine+ACSF (8)	Amplitude: Morphine+Naloxone (8)	Two-tailed unpaired t-test	$P = 0.6870$	$t_{(14)} = 0.4114$
Supplementary Fig. 8j	Morphine+ACSF(8)	Morphine+Naloxone (8)	Two-way RM ANOVA with Bonferroni post hoc analysis	$P = 0.8119$	$F_{(1, 14)} = 0.05882$
	BL			$P > 0.9999$	
	2 h			$P > 0.9999$	
	4 h			$P > 0.9999$	
	6 h			$P = 0.6759$	
	8 h			$P > 0.9999$	
	10 h			$P > 0.9999$	
Supplementary Fig. 8l	Frequency: Morphine+ACSF (8)	Frequency: Morphine+Naloxone (8)	Two-tailed unpaired t-test	$P = 0.9303$	$t_{(14)} = 0.08899$
	Amplitude: Morphine+ACSF (8)	Amplitude: Morphine+Naloxone (8)	Two-tailed unpaired t-test	$P=0.8820$	$t_{(14)} = 0.1512$
Supplementary Fig. 8n	Morphine+ACSF(8)	Morphine+Naloxone (8)	Two-way RM ANOVA with Bonferroni post hoc analysis	$P = 0.5297$	$F_{(1, 14)} = 0.4154$
	BL			$P > 0.9999$	
	2 h			$P > 0.9999$	
	4 h			$P > 0.9999$	
	6 h			$P > 0.9999$	
	8 h			$P = 0.7988$	
	10 h			$P > 0.9999$	
Supplementary Fig. 8p	Frequency: Morphine+ACSF (8)	Frequency: Morphine+Naloxone (8)	Two-tailed unpaired t-test	$P = 0.3870$	$t_{(14)} = 0.8929$

	Amplitude: Morphine+ACSF (8)	Amplitude: Morphine+Naloxone (8)	Two-tailed unpaired t-test	$P = 0.8465$	$t_{(14)} = 0.1972$
Supplementary Fig. 8r	Morphine+ACSF(8)	Morphine+Naloxone (8)	Two-way RM ANOVA with Bonferroni post hoc analysis	$P = 0.6687$	$F_{(1, 14)} = 0.1911$
	BL			$P > 0.9999$	
	2 h			$P > 0.9999$	
	4 h			$P > 0.9999$	
	6 h			$P = 0.3044$	
	8 h			$P = 0.4282$	
	10 h			$P = 0.5705$	
Supplementary Fig. 8t	Frequency: Morphine+ACSF (8)	Frequency: Morphine+Naloxone (8)	Two-tailed unpaired t-test	$P = 0.5058$	$t_{(14)} = 0.6829$
	Amplitude: Morphine+ACSF (8)	Amplitude: Morphine+Naloxone (8)	Two-tailed unpaired t-test	P=0.7852	$t_{(14)} = 0.2778$
Supplementary Fig. 9a	Morphine+EGFP (5)	Morphine+shMOR (5)	Two-tailed unpaired t-test	$P = 0.0080$	$t_{(8)} = 3.508$
Supplementary Fig. 9b	Morphine+EGFP (10)	Morphine+shMOR (10)	Two-tailed unpaired t-test	$P < 0.0001$	$t_{(18)} = 9.178$
Supplementary Fig. 9f	Frequency: Morphine+EGFP (5)	Frequency: Morphine+shMOR (5)	Two-tailed unpaired t-test	$P = 0.2910$	$t_{(8)} = 1.131$
	Amplitude: Morphine+EGFP (5)	Amplitude: Morphine+shMOR (5)	Two-tailed unpaired t-test	$P = 0.9904$	$t_{(8)} = 0.01240$
Supplementary Fig. 10b	Saline (10)	Naloxegol 30 mg/kg (10)	Two-way RM ANOVA with Bonferroni post hoc analysis	$P = 0.0165$	$F_{(3, 36)} = 3.899$

	BL			$P = 0.6650$	
	2 h			$P = 0.9657$	
	4 h			$P < 0.0001$	
	6 h			$P < 0.0001$	
	8 h			$P = 0.1131$	
	10 h			$P = 0.9885$	
Supplementary Fig. 10c	Saline (8)	Naloxegol 10 mg/kg (8)	one-way ANOVA with Bonferroni post hoc analysis	$P = 0.0003$	$F_{(3, 28)} = 8.797$
	Naloxegol 30 mg/kg (8)	Naloxegol 100 mg/kg (8)			
	Saline vs. Naloxegol 10 mg/kg			$P = 0.0220$	
	Saline vs. Naloxegol 30 mg/kg			$P = 0.0004$	
	Saline vs. Naloxegol 100 mg/kg			$P = 0.0012$	
Supplementary Fig. 10d	Saline (10)	Naloxegol 10 mg/kg (10)	one-way ANOVA with Bonferroni post hoc analysis	$P < 0.0001$	$F_{(3, 36)} = 28.08$
	Naloxegol 30 mg/kg (10)	Naloxegol 100 mg/kg (10)			
Supplementary Fig. 10e	Saline (10)	Naloxegol 30 mg/kg (10)	Two-tailed unpaired t-test	$P = 0.0025$	$t_{(18)} = 3.504$
Supplementary Fig. 10g	Frequency: Saline (8)	Frequency: Naloxegol 30 mg/kg (8)	Two-tailed unpaired t-test	$P < 0.0001$	$t_{(14)} = 5.788$
	Amplitude: Saline (8)	Amplitude: Naloxegol 30	Two-tailed unpaired t-	$P = 0.0041$	$t_{(14)} = 3.426$

		mg/kg (8)	test		
Supplementary Fig. 11d	Saline (11)	Morphine (12)	Two-tailed unpaired t-test	$P < 0.0001$	$t_{(21)} = 6.676$
Supplementary Fig. 11f	EGFP (5)	Caspase3 (5)	Two-tailed unpaired t-test	$P < 0.0001$	$t_{(8)} = 12.02$
Supplementary Fig. 11h	EGFP (8)	Caspase3 (14)	Two-tailed unpaired t-test	$P < 0.0001$	$t_{(20)} = 8.446$
Supplementary Fig. 12c	Morphine+mCherry (10)	Morphine+hM3Dq (10)	Two-way RM ANOVA with Bonferroni post hoc analysis	$P = 0.3149$	$F_{(1, 18)} = 1.069$
	BL			$P > 0.9999$	
	1 h			$P > 0.9999$	
	2 h			$P = 0.0356$	
	3 h			$P = 0.0220$	
	4 h			$P > 0.9999$	
	5 h			$P > 0.9999$	
	6 h			$P > 0.9999$	
Supplementary Fig. 12d	Morphine+mCherry (10)	Morphine+hM3Dq (10)	Two-tailed unpaired t-test	$P = 0.0226$	$t_{(18)} = 2.494$
Supplementary Fig. 12e	Morphine+ mCherry (5)	Morphine+hM3Dq (5)	Two-tailed unpaired t-test	$P = 0.0072$	$t_{(8)} = 3.580$
Supplementary Fig. 12g	Frequency: Morphine+ mCherry (6)	Frequency: Morphine+hM3Dq (6)	Two-tailed unpaired t-test	$P = 0.0035$	$t_{(10)} = 3.799$
	Amplitude: Morphine+ mCherry (6)	Amplitude: Morphine+ hM3Dq (6)	Two-tailed unpaired t-test	$P = 0.0001$	$t_{(10)} = 6.088$

Table S2. Key resource table.

REAGENT or RESOURCE	SOURCE	IDENTIFIER
**Antibodies**		
Rabbit Anti-Glutamate antibody	Sigma-Aldrich	Cat# G6642; RRID: AB_259946
Rabbit Anti-GABA antibody	Sigma-Aldrich	Cat# A2052; RRID: AB_477652
Goat Anti-ChAT antibody	Millipore	Cat# AB144P; RRID: AB_2079751
Rabbit Anti-Mu Opioid Receptor antibody	Abcam	Cat# AB134054
Rabbit Anti-TUBBIII antibody	Abcam	Cat# AB52623
Alexa 488-conjugated anti-rabbit IgG	Invitrogen	Cat# A21206; RRID: AB_2535792
Alexa 594-conjugated anti-rabbit IgG	Invitrogen	Cat# A21207; RRID: AB_141637
Alexa 594-conjugated anti-goat IgG	Invitrogen	Cat# A32758, RRID:AB_2762828
Alexa 488-conjugated anti-goat IgG	Invitrogen	Cat# A32814, RRID:AB_2534102
Mouse Anti-Neun antibody	Abcam	Cat# AB279296
Rat Anti-GFAP antibody	Abcam	Cat# AB279291
Goat Anti-Iba1 antibody	Abcam	Cat# AB289874
Mouse Anti-VgluT2 antibody	Abcam	Cat# AB216463
**Bacterial and Virus Strains**		
retro-AAV-hSyn-CRE-WPRE-hGH	BrainVTA	Cat# PT-0136
retro-AAV-hSyn-EGFP-WPRE-hGH	BrainVTA	Cat# PT-1990
rAAV-EF1α-DIO-RVG-WPRE-hGH pA	BrainVTA	Cat# PT-0023
rAAV-EF1α-DIO-H2B-mCherry-T2A-TVA-WPRE-hGH pA	BrainVTA	Cat# PT-0207
RV-EnvA-ΔG-EGFP	BrainVTA	Cat# R05001
rAAV-Ef1α-DIO-hChR2 (H134R)-mCherry	BrainVTA	Cat# PT-0002
rAAV-Ef1α-DIO-hM4D(Gi)-mCherry-WPRE-pA	BrainVTA	Cat# PT-0043
rAAV-EF1a-DIO-hM4D(Gi)-EGFP-WPREs	BrainVTA	Cat# PT-0987
rAAV-Ef1α-DIO-hM3D(Gq)-mCherry-WPRE-pA	BrainVTA	Cat# PT-0042
rAAV- Ef1α-DIO-EGFP-WPRE-Pa	BrainVTA	Cat# PT-0795
rAAV-EF1α-DIO-mCherry-WPRE-hGH pA	BrainVTA	Cat# PT-0013
rAAV-Ef1a-DIO-GCaMP6m-WPRE-pA	BrainVTA	Cat# PT-0283

rAAV-CMV-DIO-taCasp3-TEVp-WPRE-hGH polyA	BrainVTA	Cat# PT-0390
rAAV-CAG-DIO-mCherry-mCherry-WPREs	BrainVTA	Cat# PT-1529
rAAV-hSyn-CRE-mCherry-WPRE-hGH polyA	BrainVTA	Cat# PT-0407
rAAV-CMV-DIO-(EGFP-U6)-shRNA (Oprm1)-WPRE-hGH pA	BrainVTA	Cat#PT-7594
rAAV-CMV-DIO-(EGFP-U6)-shRNA(scramble)-WPRE-hGH polyA	BrainVTA	Cat#PT-2644
**Chemicals, Peptides, and Recombinant Proteins**		
Morphine	Northeast pharm Co., Ltd	Cat: 230802
Naloxone hydrochloride	Lummy Pharmaceutical Co., Ltd	Cat: D230501
Naloxegol (oxalate)	MedChemExpress	Cat: HY-A0118A
DNQX	Sigma-Aldrich	Cat# D0540; CAS:2379-57-9
Clozapine N-oxide	Sigma-Aldrich	Cat# C-0832
DAPI	Biosharp	Cat# BL105A
Fluoro-Gold	Fluorochrome	Cat# Fluoro-gold;<br>RRID: AB_2314408
Tetrodotoxin	Sigma-Aldrich	Cat# 554412
Picrotoxin	Sigma-Aldrich	Cat# R284556
D-AP5	Sigma-Aldrich	Cat# A8054
Activated charcoal	Sigma-Aldrich	Cat# 901931
Gum acacia	Sigma-Aldrich	Cat# G9752
**Experimental Models: Organisms/Strains**		
Mouse: wild type C57BL/6J	Charles River	N/A
Mouse: *Vglut2-ires-cre*: B6J.129S6(FVB)-Slc17a6tm2(cre)Lowl/MwarJ	Jackson Laboratory	028863;<br>RRID: IMSR_JAX:028863
Mouse: *ChAT-Cre:* B6;129S6-Chattm2(cre)Lowl/J	Jackson Laboratory	006410
Mouse: *Ai14*: B6.Cg-Gt(ROSA)26 Sortm14(CAG-tdTomato)Hze/J	Jackson Laboratory	007914;<br>RRID: IMSR_JAX:007914
**Software and Algorithms**		
Fiji software	Schindelin et al., 2012	https://imagej.net/software/fiji/downloads
Illustrator CS6	Adobe	https://www.adobe.com/products/illustrator.html
GraphPad Prism 8	GraphPad Software	https://www.graphpad.com/scientific-software/

Clampfit 10.7	Molecular Devices	https://www.moleculardevices.com/products/axon-patch-clamp-system/acquisition-and-analysis-software/pclamp-software-suite
CaImAn-MATLAB	MATLAB	https://github.com/flatironinstitute/CaImAn-MATLAB
ZEN2012	Zeiss	https://www.zeiss.com/microscopy/en/products/software/zeiss-zen-desk.html#downloads
IMARIS 9.6.2	Bitplane	https://imaris.oxinst.com/packages
LabChart 8 software	AD Instruments	https://www.adinstruments.com/support/software#labchart
Inscopix Data Processing Software v1.3.1	Inscopix	https://iq.inscopix.com/software
**Other**		
Optogenetic fibers	Inper	N/A
Cannula	RWD	N/A

206
