# Supplementary material for: A brain-to-small intestine circuit mediates morphine-induced constipation in male mice
Source: Nat Commun. 2025 Dec 23;17:1023. doi: 10.1038/s41467-025-67765-7 (PMC12847740; doi:10.1038/s41467-025-67765-7)
Supplement: Supplementary file 2 — Description of Additional Supplementary Files [file 41467_2025_67765_MOESM2_ESM.pdf]

Description of Additional Supplementary Files:

Supplementary Movie 1: 3D visualization of the mCherrylabeled DMV^{Ach} neuronal terminals in the small intestine.

Supplementary Movie 2: Microendoscopic calcium imaging for GCaMP6m-expressing DMV^{Ach} neurons in mice before and after morphine treatment.

Supplementary Movie 3: Microendoscopic calcium imaging for DMV-projecting PVN neurons in mice before and after morphine treatment.
