# Supplementary material for: A brain-to-small intestine circuit mediates morphine-induced constipation in male mice
Source: Nat Commun. 2025 Dec 23;17:1023. doi: 10.1038/s41467-025-67765-7 (PMC12847740; doi:10.1038/s41467-025-67765-7)
Supplement: Supplementary file 6 — Reporting Summary [file 41467_2025_67765_MOESM6_ESM.pdf]

nature portfolio

Corresponding author(s): Xiaoqing Chai, Zhi Zhang, Sheng Wang, Peng Cao

Last updated by author(s): Nov 24, 2025

# Reporting Summary

Nature Portfolio wishes to improve the reproducibility of the work that we publish. This form provides structure for consistency and transparency in reporting. For further information on Nature Portfolio policies, see our Editorial Policies and the Editorial Policy Checklist.

## Statistics

For all statistical analyses, confirm that the following items are present in the figure legend, table legend, main text, or Methods section.

| n/a | Confirmed | |
|---|---|---|
| ☐ | ☒ | The exact sample size (*n*) for each experimental group/condition, given as a discrete number and unit of measurement |
| ☐ | ☒ | A statement on whether measurements were taken from distinct samples or whether the same sample was measured repeatedly |
| ☐ | ☒ | The statistical test(s) used AND whether they are one- or two-sided<br>*Only common tests should be described solely by name; describe more complex techniques in the Methods section.* |
| ☒ | ☐ | A description of all covariates tested |
| ☐ | ☒ | A description of any assumptions or corrections, such as tests of normality and adjustment for multiple comparisons |
| ☐ | ☒ | A full description of the statistical parameters including central tendency (e.g. means) or other basic estimates (e.g. regression coefficient) AND variation (e.g. standard deviation) or associated estimates of uncertainty (e.g. confidence intervals) |
| ☐ | ☒ | For null hypothesis testing, the test statistic (e.g. *F*, *t*, *r*) with confidence intervals, effect sizes, degrees of freedom and *P* value noted<br>*Give P values as exact values whenever suitable.* |
| ☒ | ☐ | For Bayesian analysis, information on the choice of priors and Markov chain Monte Carlo settings |
| ☒ | ☐ | For hierarchical and complex designs, identification of the appropriate level for tests and full reporting of outcomes |
| ☐ | ☒ | Estimates of effect sizes (e.g. Cohen's *d*, Pearson's *r*), indicating how they were calculated |

*Our web collection on statistics for biologists contains articles on many of the points above.*

## Software and code

Policy information about availability of computer code

| | |
|---|---|
| Data collection | For slice physiological recording, the current and voltage signals were recorded with MultiClamp 700B and Clampex 10 data acquisition software (Molecular Device, USA). Images were captured on a Zesis LSM980 confocal microscope (Germany). For intestinal vagus nerve firing rate and intestinal motility recording. the signals were recorded by the Labchart 8 software (AD instruments, Australia). For microendoscopic imaging, the raw data were processed using Mosaic (Inscopix, USA) |
| Data analysis | For slice physiological recording, the data were analyzed using Clampfit 10.3 (Molecular devices, USA). GraphPad Prism 8 (Graph Pad software, inc, USA) was used for the statistical analyses. Imaging data were processed and analyses using ZEN 2012, Image J software (Fiji edition, NIH), LitScan or Imaris (Bitplane). Labchart 8 software was used to conduct offline analyses of data from intestinal vagus nerve firing rate and intestinal motility recordings. For microendoscopic imaging, we used custom MATLAB (Mathworks, 2019b) for further analysis. |

For manuscripts utilizing custom algorithms or software that are central to the research but not yet described in published literature, software must be made available to editors and reviewers. We strongly encourage code deposition in a community repository (e.g. GitHub). See the Nature Portfolio guidelines for submitting code & software for further information.

## Data

Policy information about availability of data

All manuscripts must include a data availability statement. This statement should provide the following information, where applicable:

- Accession codes, unique identifiers, or web links for publicly available datasets
- A description of any restrictions on data availability
- For clinical datasets or third party data, please ensure that the statement adheres to our policy

All data necessary to understand and assess the conclusions of this study are available in the main text or the supplementary materials. There are no restrictions on data availability in the manuscript. Source data are provided with this paper.

## Research involving human participants, their data, or biological material

Policy information about studies with human participants or human data. See also policy information about sex, gender (identity/presentation), and sexual orientation and race, ethnicity and racism.

| | |
|---|---|
| Reporting on sex and gender | N/A |
| Reporting on race, ethnicity, or other socially relevant groupings | N/A |
| Population characteristics | N/A |
| Recruitment | N/A |
| Ethics oversight | N/A |

Note that full information on the approval of the study protocol must also be provided in the manuscript.

# Field-specific reporting

Please select the one below that is the best fit for your research. If you are not sure, read the appropriate sections before making your selection.

☒ Life sciences ☐ Behavioural & social sciences ☐ Ecological, evolutionary & environmental sciences

For a reference copy of the document with all sections, see nature.com/documents/nr-reporting-summary-flat.pdf

# Life sciences study design

All studies must disclose on these points even when the disclosure is negative.

| | |
|---|---|
| Sample size | No statistical methods were used to pre-determine sample sizes but our sample sizes are similar to those reported in previous publications (PMID: 39984470, PMID: 40240334, PMID: 37592008). |
| Data exclusions | For all experiments, mice with signs of infection/bleeding/unhealthy conditions after the surgeries were excluded for behavioral tests and mice with missed viral injections or implantation targets, as described by brain atlas, were not included in experimental analyses. |
| Replication | All experiments are replicated multiples times with independent mice, and at least two people independently analyzed time points for the behavioral events. Numbers of replicates (n) are indicated in the figure legends and Supplementary Table S1. |
| Randomization | The animals in the experiments were randomized assigned. |
| Blinding | All investigators were blinded to group allocation during data collection and analysis. |

# Reporting for specific materials, systems and methods

We require information from authors about some types of materials, experimental systems and methods used in many studies. Here, indicate whether each material, system or method listed is relevant to your study. If you are not sure if a list item applies to your research, read the appropriate section before selecting a response.

## Materials & experimental systems

| n/a | Involved in the study |
|---|---|
| | x Antibodies |
| x | Eukaryotic cell lines |
| x | Palaeontology and archaeology |
| | x Animals and other organisms |
| x | Clinical data |
| x | Dual use research of concern |
| x | Plants |

## Methods

| n/a | Involved in the study |
|---|---|
| x | ChIP-seq |
| x | Flow cytometry |
| x | MRI-based neuroimaging |

# Antibodies

| | |
|---|---|
| Antibodies used | For immunohistochemistry the following antibodies were used: rabbit antiglutamate (1:500, G6642, Sigma), rabbit anti-GABA (1:500, A2052, Sigma), goat anti-ChAT (1:500, AB144P, Millipore), rabbit anti-mu opioid receptor (1:500, AB134054, Abcam), rabbit anti-TUBBIII (1:500, AB52623, Abcam), mouse anti-neun (1:500, AB279296, Abcam), rat anti-GFAP (1:500, AB279291, Abcam), goat anti-Iba1 (1:500, AB289874, Abcam), mouse anti-VgluT2 (1:500, AB216463, Abcam). |
| Validation | All antibodies used in this study were obtained from commercial suppliers and were validated by the manufacturers for their specific application (immunohistochemistry). The validation is reported on their websites. |

# Animals and other research organisms

Policy information about studies involving animals; ARRIVE guidelines recommended for reporting animal research, and Sex and Gender in Research

| | |
|---|---|
| Laboratory animals | The experiments used adult (8-10 weeks) C57BL/6J (Charles River), ChAT-Cre (Jackson Labs, 006410), VgluT2-Cre (Jackson Labs, 028862), Ai14 (RCL-tdT) (Jackson Labs, 007914) lines of male mice. |
| Wild animals | The study did not involve wild animals. |
| Reporting on sex | Male animals were used in all experiments. |
| Field-collected samples | This study did not involve samples collected from the field. |
| Ethics oversight | All animal protocols were approved by the Animal Care and Use Committee of the University of Science and Technology of China. |

Note that full information on the approval of the study protocol must also be provided in the manuscript.

# Plants

| | |
|---|---|
| Seed stocks | N/A |
| Novel plant genotypes | N/A |
| Authentication | N/A |
